# Complete chloroplast genomes of six neotropical palm species, structural comparison, and evolutionary dynamic patterns

**DOI:** 10.1038/s41598-023-44631-4

**Published:** 2023-11-23

**Authors:** Ana Flávia Francisconi, Jonathan Andre Morales Marroquín, Luiz Augusto Cauz-Santos, Cássio van den Berg, Kauanne Karolline Moreno Martins, Marcones Ferreira Costa, Doriane Picanço-Rodrigues, Luciano Delmodes de Alencar, Cesar Augusto Zanello, Carlos Augusto Colombo, Brenda Gabriela Díaz Hernández, Danilo Trabuco Amaral, Maria Teresa Gomes Lopes, Elizabeth Ann Veasey, Maria Imaculada Zucchi

**Affiliations:** 1 Programa de Pós-Gradução em Genética e Biologia Molecular, Universidade Estadual de Campinas, R. Monteiro Lobato, 255-Barão Geraldo, Campinas, São Paulo CEP 13083-862 Brazil; 2 Department of Botany and Biodiversity Research, University of Vienna, Rennweg 14, 1030 Wien, Austria; 3 Departamento de Ciências Biológicas, Universidade Estadual de Feira de Santana, Av. Transnordestina S/N-Novo Horizonte, Feira de SantanaFeira de Santana, Bahia CEP 44036-900 Brazil; 4 Universidade Federal do Piauí, BR-343 Km 3.5, Floriano, Piauí CEP 64808-605 Brazil; 5 Departamento de Biologia, Universidade Federal do Amazonas, Avenida Gen. Rodrigo Octávio Jordão Ramos, 3000-Coroado I-Campus Universitário-Senador Arthur Virgílio Filho-Setor Sul, Bloco H, Manaus, Amazonas CEP 69077-000 Brazil; 6 Instituto Agronômico, Av. Theodureto de Almeida Camargo, 1500, Campinas, São Paulo CEP 13075-630 Brazil; 7 Departamento de Biologia, Centro de Ciências Humanas e Biológicas, Universidade Federal do ABC, Avenida dos Estados, 5001, Santo André, São Paulo CEP 09040-040 Brazil; 8 Faculdade de Ciências Agrárias, Universidade Federal do Amazonas, Avenida Rodrigo Otávio Ramos, 3000-Bairro Coroado, Manaus, Amazonas CEP 69077-000 Brazil; 9 Departamento de Genética, Escola Superior de Agricultura “Luiz de Queiroz”, Universidade de São Paulo, Avenida Pádua Dias, 11-Bairro São Dimas, Piracicaba, São Paulo, CEP 13418-900 Brazil; 10 Agência Paulista de Tecnologia dos Agronegócios (APTA), Polo Centro Sul, Rodovia SP 127 Km 30, CP 28, Piracicaba, São Paulo CEP 13400-970 Brazil

**Keywords:** Evolutionary biology, Genome, Plant genetics, Genetic markers, Chloroplasts

## Abstract

The Arecaceae family has a worldwide distribution, especially in tropical and subtropical regions. We sequenced the chloroplast genomes of *Acrocomia intumescens* and *A. totai*, widely used in the food and energy industries; *Bactris gasipaes*, important for palm heart; *Copernicia alba* and *C. prunifera*, worldwide known for wax utilization; and *Syagrus romanzoffiana*, of great ornamental potential. *Copernicia* spp. showed the largest chloroplast genomes (*C. prunifera:* 157,323 bp and *C. alba*: 157,192 bp), while *S. romanzoffiana* and *B. gasipaes* var*. gasipaes* presented the smallest (155,078 bp and 155,604 bp). Structurally, great synteny was detected among palms. Conservation was also observed in the distribution of single sequence repeats (SSR). *Copernicia* spp. presented less dispersed repeats, without occurrence in the small single copy (SSC). All RNA editing sites were C (cytidine) to U (uridine) conversions. Overall, closely phylogenetically related species shared more sites. Almost all nodes of the phylogenetic analysis showed a posterior probability (PP) of 1.0, reaffirming the close relationship between *Acrocomia* species*.* These results elucidate the conservation among palm chloroplast genomes, but point to subtle structural changes, providing support for the evolutionary dynamics of the Arecaceae family.

## Introduction

The Arecaceae family comprises 188 genera and 2585 species. Its distribution is cosmopolitan and worldwide, and it is concentrated mainly in tropical and subtropical regions^1–3^. Commonly known as palms, approximately > 800 species are distributed throughout the Neotropical region and, in South America alone, 437 species occur, belonging to 50 genera, among which 18 are endemic^4^. Palms are one of the dominant species groups in the tropical rainforest (TRF). Also, the Arecaceae family presents high species richness, and is a key component in the evolution and diversification of hyper diversity in the TRF biome^5,6^. Palms are part of the TRF ecosystem’s services. For example, they are a base resource for frugivory and pollinators, and may have influenced the diversification of dependent animal groups. Palms also have a shared history with ancestral human groups providing food, construction materials, fuel, and ornamentals^7,8^. According to Huang et al.^9^, Arecaceae species constitute the third most economically important family in the world.

Among palm species, the genus *Acrocomia* Mart. (subtribe Bactridinae, tribe Cocoseae) is widely distributed in the Neotropics and is popularly known as Macaúba (Macaw palm) or Coyol. *Acrocomia* includes eight species, of which *Acrocomia aculeata* (Jacq.) Lodd. Ex Mart., *A. intumescens* Drude, and *A. totai* Mart. have great economic interest, considering that all vegetative structures can be used^10^. The three species have high oil content and are promising for the energy and food industries, thus presenting native alternative sources for food and biodiesel production^11,12^. Besides, new products can be exploited, such as *A. totai*, for example, which is commonly used as food in its habitat^12^.

*Bactris gasipaes* Kunth. (Bactridinae), commonly known as Pupunha (Peach palm), was an important pre-Columbian subsistence product, used for fruit consumption and its stipe woody structures for hunting, fishing, and agricultural equipment. Nowadays, the fruit is commercialized in its natural state or traditionally used in regional dishes at Amazonian markets. *B. gasipaes* is essential to supply the production chain of high-quality heart-of-palm^13^. Another Neotropical palm genus is *Copernicia* (subtribe Livistoniinae, tribe Trachycarpeae), comprising 21 species mainly distributed in North America, Greater Antilles, with only three occurring in South America^14^. In Brazil, *Copernicia alba* Morong ex Morong &. Britton, popularly known as Carandá (Caranda palm), is economically important due to the quality of its wood, the consumption of heart-of-palm in the food industry, and the ornamental potential^15^. *Copernicia prunifera* (Mill.) H.E.Moore, known as Carnaúba (Carnaúba palm), is a worldwide source of quality carnauba wax^16,17^ and can also be used in cosmetics, skin care, and candy coating^18^. Both palms are endemic to Brazilian biomes, the Pantanal and the Caatinga, respectively. *Syagrus romanzoffiana* (Cham.) Glassman (subtribe Attaleinae, tribe Cocoseae), popularly known as Coqueiro or Jerivá (Queen palm or Cocos palm), has ornamental potential and fruits with high nutritional content, used as food sources for animals. The oil obtained from this species is applicable to the food, cosmetics, pharmaceutical, and biodiesel industries^19^. Finally, it is important to highlight that the six chosen Neotropical palm species also provide key ecosystem services that ensure welfare for indigenous people in South America^8,20,21^. Due to their growing economic importance and traditional use, continued efforts to obtain genetic and genomic resources are essential to gather information about these species and thus plan their uses in a sustainable way.

The rapid development of next-generation sequencing (NGS) technology has made it cheaper and more accessible to obtain complete chloroplast sequences, even for an increasing number of non-models species^22–25^. The chloroplast is a semi-autonomous organelle in plant cells, with its own genetic information, a complete genetic system, and its genome is typically uniparentally inherited in the absence of recombination^26^. The chloroplast genome usually has four parts: two single copy regions, a large one (LSC), a small one (SSC), and a pair of inverted regions (IRs)^27^. This genome consists basically of long circular or linear molecules (120–180 kb), containing 120–130 genes with functions for photosynthesis, transcription, and translation. The genome structure and gene content are, in general, highly conserved in land plants^28^, including palm species^29^.

For palm species occurring in Brazil, 12 complete chloroplast genomes have been reported^2,29–36^. These genomes are used for structural comparisons, phylogenetic inferences, the determination of RNA editing sites, and the identification of genomic regions with different repeats and polymorphisms. Among the advantages of elucidating new palm chloroplast genomes are increasing our understanding of evolutionary processes in Arecaceae and deciphering phylogenetic relationships between closely related taxa^28^. Furthermore, with the available sequences, it will be possible to identify repetitive sequences and regions with high polymorphism and screen for molecular markers. These resources are extremely important for assessing genetic structure and diversity in natural populations^34^.

Considering the importance and necessity of increasing genomic information about these Neotropical palm species, here we report the complete chloroplast genomes of *A. intumescens, A. totai, B. gasipaes* var*. gasipaes, C. alba, C. prunifera* and *S. romanzoffiana*. With the complete chloroplast genomes, we were able to investigate: (1) gene content; (2) structural comparisons and the synteny level with other chloroplast genomes available from Arecaceae species; (3) single sequence repeats and dispersed repeats; (4) RNA editing sites and their conservancy among the six palm species; and (5) the Arecaceae family evolution based on a phylogenomic study with six new complete chloroplast genomes.

## Results

### Organization and gene features of the six palms chloroplast genomes

**Figure 1:**
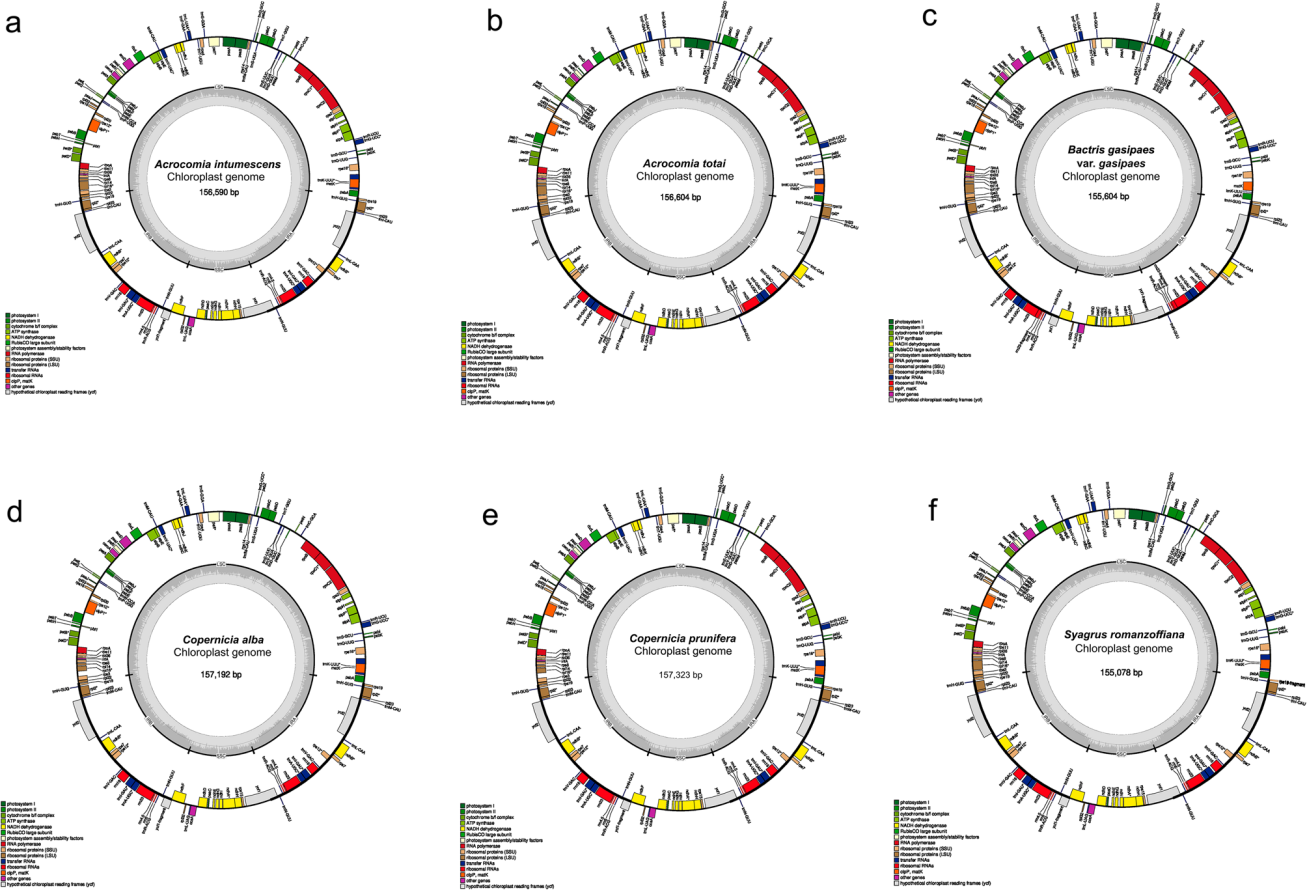
Gene map of (**a**) *Acrocomia intumescens*, (**b**) *A. totai,* (**c**) *Bactris gasipaes* var. *gasipaes*, (**d**) *Copernicia alba,* (**e**) *C. prunifera*, and (**f**) *Syagrus romanzoffiana* chloroplast genomes. Genes represented inside the large circle are oriented clockwise, and those outside are oriented counterclockwise. The distinct colors represent functional groups, and the darker gray in the inner circle indicates the GC content. The quadripartite structure is also reported as: *LSC* large single copy, *SSC* small single copy, *IRA* inverted repeat A, *IRB* inverted repeat B.

The chloroplast genomes of the six palm species had the typical quadripartite structure with the presence of two single copies (large single copy—LSC, small single copy—SSC; Fig. 1a–f), and two inverted repeat regions (IRA and IRB). The species from the *Copernicia* genus had the largest chloroplast genome size (*C. prunifera*: 157,323 bp and *C. alba*: 157,192 bp; Fig. 1d,e, Table 1), and *S. romanzoffiana* had the smallest size (155,078 bp; Fig. 1f). The species from the genus *Copernicia* also displayed the longest LSC (*C. alba*: 86,430 bp and *C. prunifera*: 86,264 bp). However, *Acrocomia intumescens* presented the largest SSC (17,522 bp). Finally, the species *A. intumescens*, *A. totai*, and *B. gasipaes* var. *gasipaes* showed IRs with more nucleotides (bp) than the species of the genus *Copernicia* and *S. romanzoffiana*. All species had GC content (%) between 37.2 and 38.0, with a similar distribution (%) of GC content in the four regions of the complete chloroplast genome (Table 1).

**Table 1:** Gene features in the *Acrocomia intumescens*, *A. totai, Bactris gasipaes* var. *gasipaes, Copernicia alba, C. prunifera*, and *Syagrus romanzoffiana* chloroplast genomes according to each respective category.

Gene features	*A. intumescens*	*A. totai*	*B. gasipaes* var. *gasipaes*	*C. alba*	*C. prunifera*	*S. romanzoffiana*
NCBI accession number	OQ129926	OQ129927	OQ129928	OQ129929	OQ129930	OQ129931
Total cpDNA size (bp)	156,590	156,604	155,604	157,192	157,323	155,078
LSC length (bp)	85,058	85,060	84,193	86,430	86,264	84,730
SSC length (bp)	17,522	17,360	17,362	17,440	17,475	17,473
IR length (bp)	27,068	27,092	27,024	26,661	26,792	26,437
Total GC content (%)	37.4	37.4	37.5	37.2	37.2	38.0
LSC GC content (%)	35.5	35.5	35.5	35.1	35.2	35.5
SSC GC content (%)	31.2	31.2	31.2	30.8	30.8	31.1
IR GC content (%)	42.5	42.5	42.6	42.5	42.6	42.7
Total number of genes	131	131	131	131	131	130
Protein-coding genes	85	85	85	85	85	84
rRNA genes	8	8	8m	8	8	8
tRNA genes	38	38	38	38	38	38

*SSC* small single copy, *LSC* large single copy, *IRA* inverted repeat A, *IRB* inverted repeat B.

The species presented variation in the number of genes (130–131, considering duplicate copies; Table 1). By comparing the structures of the six chloroplast genomes, it is possible to identify that *C. alba* and *C. prunifera* had two copies of the *trnM*—*CAU* and one copy of the *trnG*—*UCC*, instead of the two *trnI*—*CAU* and one *trnG*—*GCC*, respectively, that were present in the four chloroplast genomes of the species from the subfamily Arecoideae, analyzed in this study (Fig. 1). Also, *S. romanzoffiana* presented one gene reduction when compared with the other species of the subfamily Arecoideae, *rps19*, which in this case was presented as a fragment (Fig. 1).

### The chloroplast genome structures and comparative analyses among species from different genera

**Figure 2:**
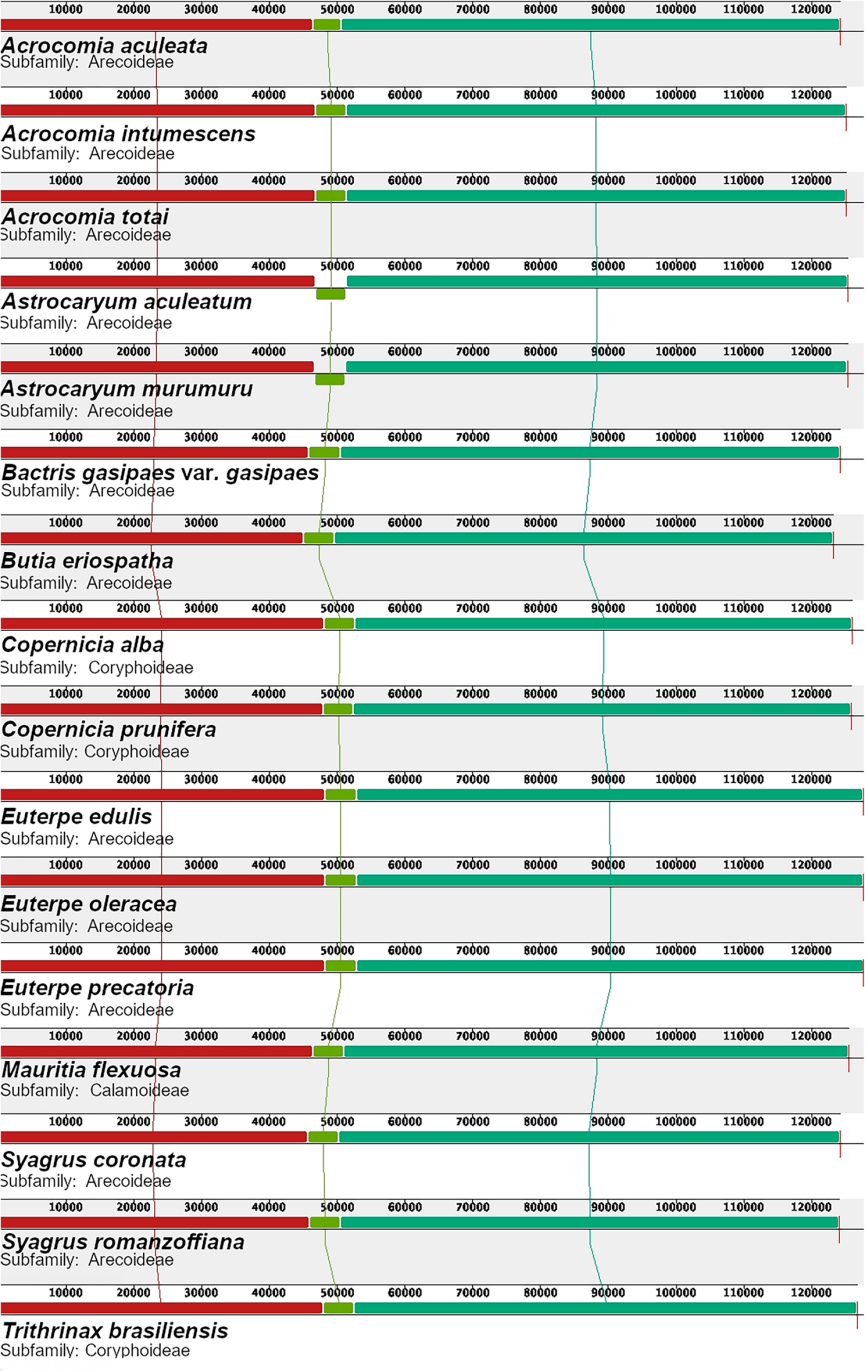
Synteny and divergence in the small single copy (SSC) size detected in Arecaceae chloroplast genomes using the Mauve multiple-genome alignment program. A sample of 16 different chloroplast genomes is shown. Color bars indicate syntenic blocks, and the lines indicate the correspondence between them. Blocks on the top row are in the same orientation, while blocks on the bottom row are in the opposite orientation.

The six Brazilian palm chloroplast genomes annotated in this study showed a high level of synteny in their structures (Fig. 2). Although the species are from different subfamilies (*C. alba*, *C. prunifera*, and *Trithrinax brasiliensis* Mart., subfamily Coryphoideae; *Mauritia flexuosa* L.f., subfamily Calamoideae; and the others from the subfamily Arecoideae), few structural rearrangements were observed. This structural conservation was also noted in the comparison of palm chloroplast genomes with five subfamilies (see Supplementary Fig. S1). The most notable change was in *Astrocaryum aculeatum* G. Mey. and *A. murumuru* Mart., which showed flip-flop recombination (in lime green; Fig. 2)^32^. As already pointed out, a significant dissimilarity among the species of the subfamily Arecoideae that occur in Brazil can be identified in the length of the LSC, between 40,000 and 50,000 bp^36^. Furthermore, between the species *C. alba* and *C. prunifera* of the subfamily Coryphoideae, it was also possible to observe a reduction in LSC size (Table 1, Fig. 2). Among the new six palm chloroplast genomes, *S. romanzoffiana* had the smallest SSC length, which was also noted in the structure of *S. coronata* (Mart.) Beec.

**Figure 3:**
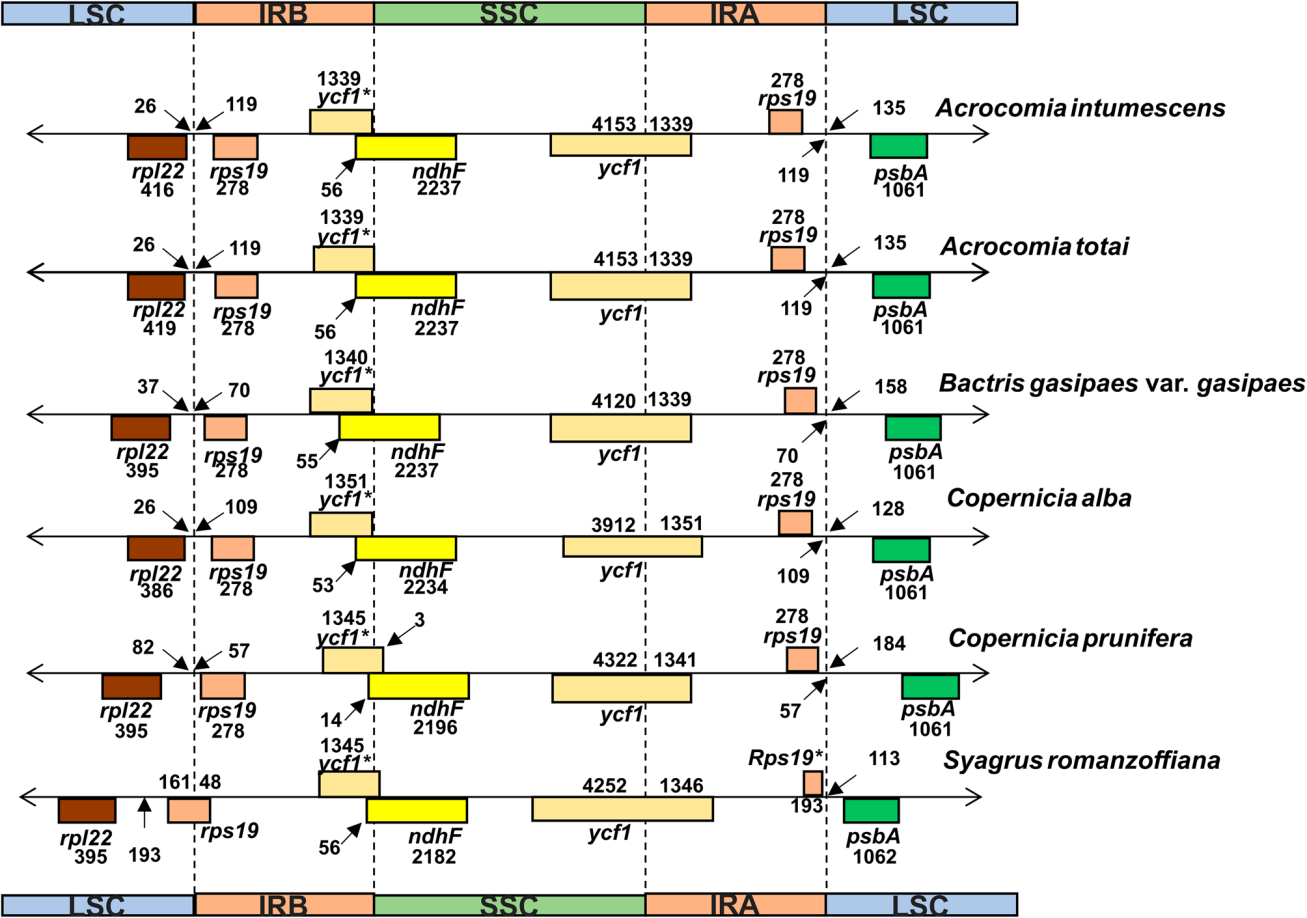
Comparison of the inverted repeats A and B (IRA and IRB) borders among Brazilian palms. The numbers indicate the lengths of IGSs, genes, and spacers between IR-LSC and IR-SSC junctions. The ycf1* and rps19* genes have incomplete CDSs. *LSC* large single copy, *SSC* small single copy.

Expansions/contractions can be observed throughout all the chloroplast genome structures of *C. alba* and *C. prunifera,* even though they are species of the same genus (Fig. 3). *C. prunifera* showed a shift in *rpl22* and *rps19* genes at the LSC/IRB margin compared to *C. alba*. The same shift can also be found in *ycf1* genes between IRB/LSC and SSC/IRA, and in *rps19* and *psbA* genes between IRA/LSC. Another very divergent species with expansions/contractions in the IR was *S. romanzoffiana*. The species presented the *rps19* gene between the LSC/IRB, while in the other analyzed palms, this gene was located completely in the IRB. In addition, the copy of *rps19* is transformed into a fragment at the boundary between the IRA/LSC.

### SSR and dispersed repeats in the chloroplast genome structures

**Figure 4:**
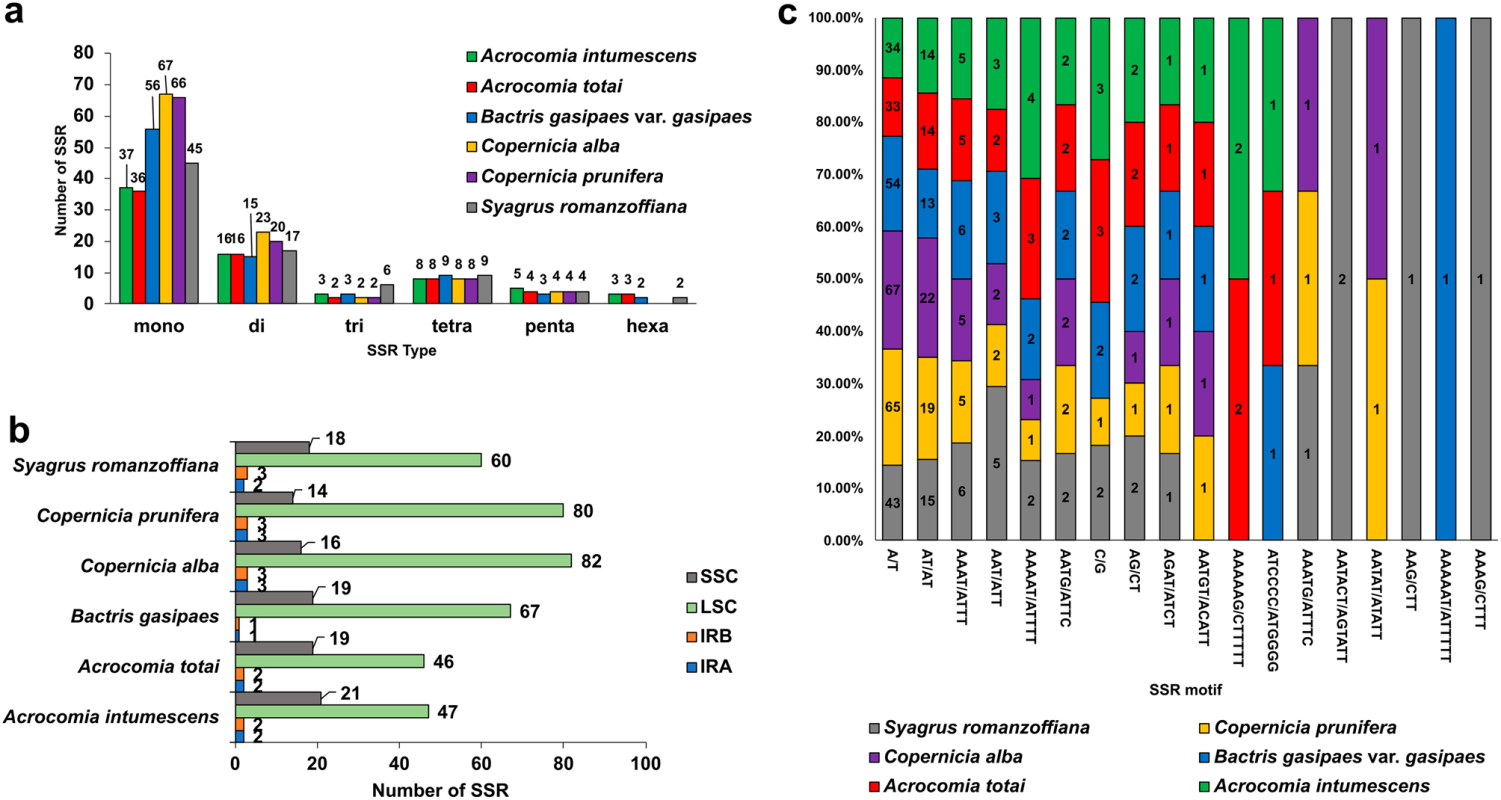
Distribution, classification, and motifs of single sequence repeats (SSR) in the chloroplast genomes of *Acrocomia intumescens, A. totai, Bactris gasipaes* var. *gasipaes, Copernicia alba, C. prunifera*, and *Syagrus romanzoffiana.* (**a**) Number of SSR types (mono-, di-, tri-, tetra-, penta-, and hexanucleotides) present in the six chloroplast genomes; (**b**) number of SSR in the different chloroplast genome regions; (**c**) number of different SSR motifs distributed in the six chloroplast genomes. *SSC* small single copy, *LSC* large single copy, *IRA* inverted repeat A, *IRB* inverted repeat B.

Among the six palm species, a total of 516 simple sequence repeats (SSRs) were identified (Fig. 4a, Supplementary Table S1). Seventy-two and 69 SSRs were identified in *A. intumescens* and *A. totai*, respectively. In *B. gasipaes* var. *gasipaes,* 88 SSRs were found. A higher amount of SSRs was observed in *C. alba* and *C. prunifera* (104 and 100 SSRs, respectively). *S. romanzoffiana* presented 83 SSRs in its chloroplast genome. All six palms had a higher number of mononucleotides of the SSR type, followed by di- and tetranucleotides, mostly concentrated in the LSC region of their chloroplast genome (Fig. 4b, Supplementary Table S1). Also, all species presented the motifs A/T, AT/AT, AAAT/ATTT, AAT/ATT, AAAAT/ATTTT, AATG/ATTC, AG/CT, and AGAT/ATCT. Some motifs were unique to certain palm species, such as AATACT/AGTATT, AAG/CTT, and AAAG/CTT, occurring only in *S. romanzoffiana*, and AAAAAT/ATTTTT, appearing only in *B. gasipaes* (Fig. 4c).

**Figure 5:**
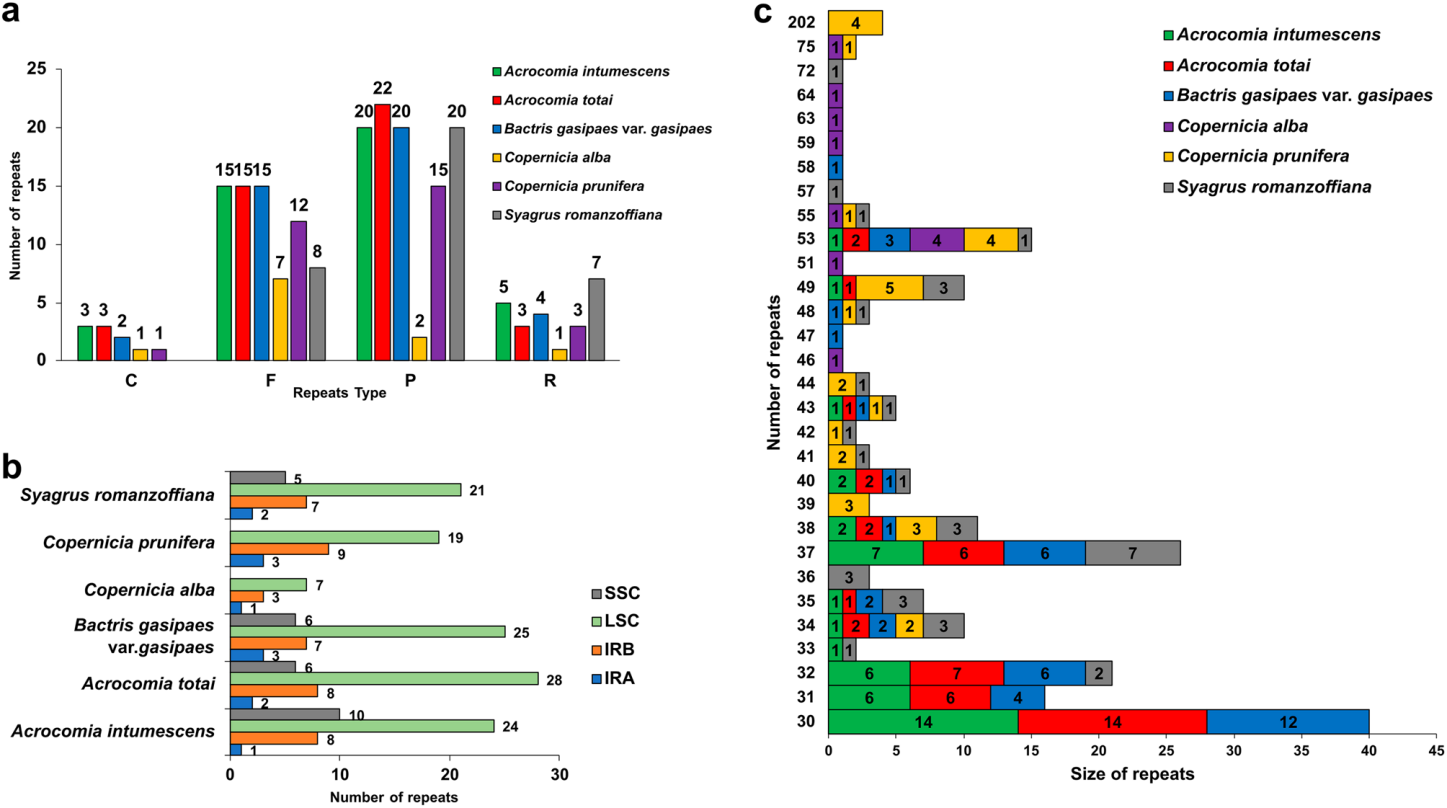
Distribution and classification of dispersed repeats in the chloroplast genomes of *Acrocomia intumesces, A. totai, Bactris gasipaes* var. *gasipaes, Copernicia alba, C. prunifera*, and *Syagrus romanzoffiana*. (**a**) Frequency distribution of different types of repeats; (**b**) number of dispersed repeats present in different chloroplast genome regions; (**c**) number of dispersed repeat sizes among the six palm species. *F* forward, *P* palindrome, *R* reverse, *C* complement, *SSC* small single copy, *LSC* large single copy, *IRA* inverted repeat A, *IRB* inverted repeat B.

The total number of dispersed repeats (F = forward, P = palindrome, R = reverse, and C = complement) was very similar in *A. intumescens* (43), *A. totai* (43), and *B. gasipaes* var. *gasipaes* (41; Supplementary Table S2, Fig. 5a). The smallest values of these repeats were observed in *S. romanzoffiana* (35), *C. prunifera* (31), and *C. alba* (11). *A. intumescens, A. totai*, and *B. gasipaes* also presented a similar distribution of repeats with a higher concentration of palindromes (P; 22–20), followed by the forward type (F; 15). Likewise, *C. prunifera* and *S. romanzoffiana* showed higher concentrations of the palindrome (15 and 20, respectively) and forward types (12 and 8, respectively). Inversely, *C. alba* had a higher number of forward (seven repeats) types, followed by palindrome types (two repeats; Fig. 5a). All studied species presented the highest number of dispersed repeats concentrated in the LSC region (Fig. 5b), while only *C. alba* and *C. prunifera* showed any dispersed repeats in the SSC region of the chloroplast genome. *A. intumescens*, *A. totai*, and *B. gasipaes* had the highest number of dispersed repeats with a size of 30 bp (14, 14, and 12, respectively; Fig. 5c). *C. alba* and *C. prunifera* had the greatest number of repeats with longer lengths, 53 bp (four repeats, *C. alba*) and 49 bp (5 repeats, *C. prunifera*). *S. romanzoffiana*, on the other hand, had the highest number of repeats of an intermediate size of 37 bp (7 repeats).

### Prediction of RNA editing sites in the chloroplast genes of the six palm species

**Figure 6:**
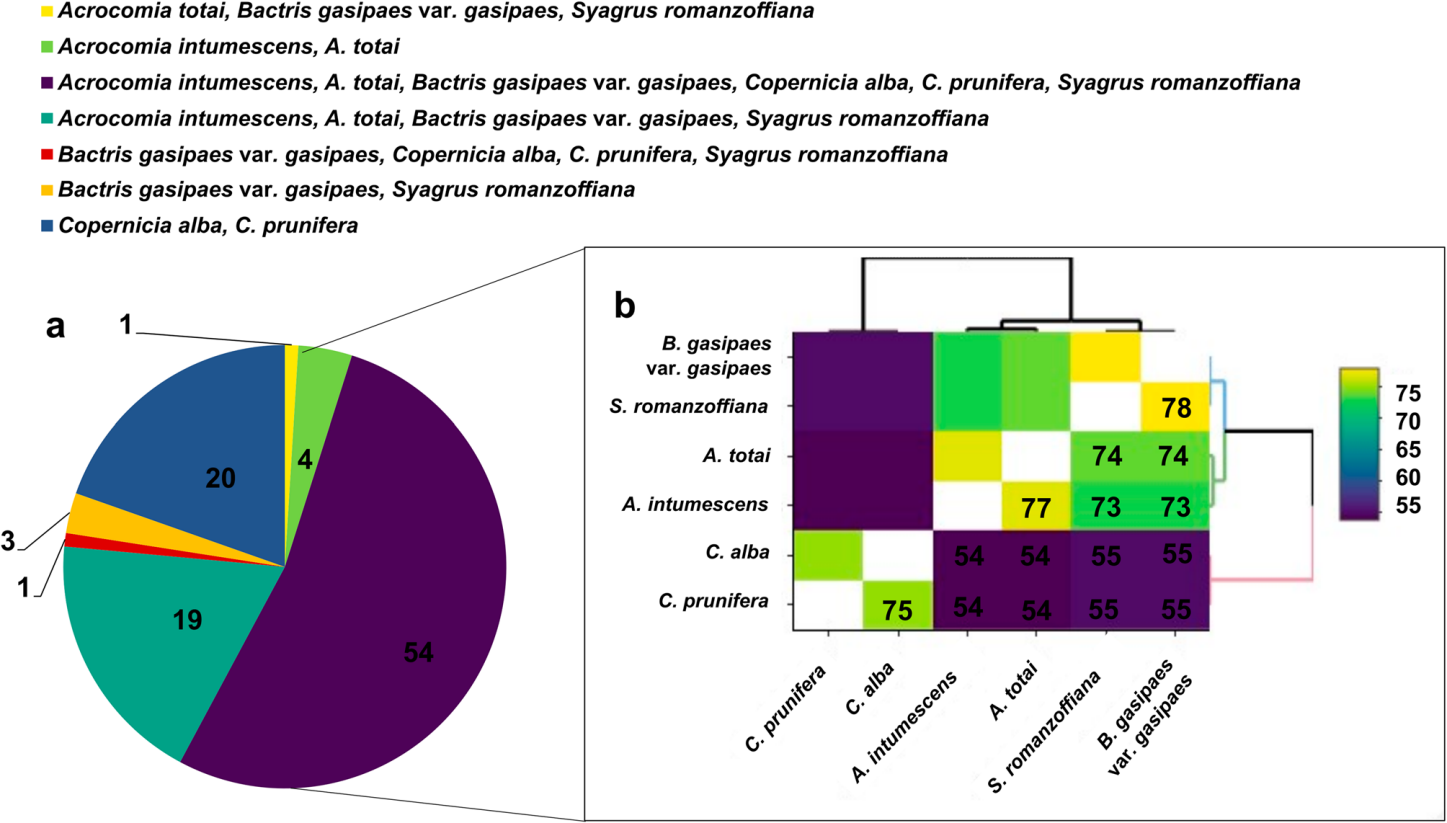
RNA editing sites shared by the species *Acrocomia intumescens, A. totai, Bactris gasipaes* var. *gasipaes, Copernicia alba, C. prunifera*, and *Syagrus romanzoffiana*. (**a**) Number of RNA editing sites shared among the six species; (**b**) Pairwise comparison of RNA editing sites found between the species.

Considering all changes, such as nucleotide and amino acid positions and conversions, 102 RNA editing sites were identified and shared among the six palm species (Fig. 6a). All RNA editing sites analyzed showed conversion of the nucleotides from cytidine (C) to uridine (U; Supplementary Table S3). The conversion occurred at the second (79.41%) and first (20.59%) codon positions. From the 102 RNA editing sites, 45 corresponded to changes from serine (S) to leucine (L), followed by 15 changes from proline (P) to leucine (L), and from histidine (H) to tyrosine (Y) (Supplementary Fig. S2a). Thus, the conversions resulted in 82.35% hydrophilic to hydrophobic amino acids (Supplementary Fig. S2b).

Among the 102 RNA editing sites, the six species shared 54 conversions. Between *C. alba* and *C. prunifera*, 20 conversions occurred exclusively (Fig. 6a). Also, among *A. intumescens*, *A. totai*, *B. gasipaes* var. *gasipaes*, and *S. romanzoffiana*, 19 conversions were observed. The number of RNA editing sites, considering pairwise species analyses, resulted in *S. romanzoffiana* × *B. gasipaes* sharing 78 sites, followed by *A. intumescens* × *A. totai* (77), and *C. alba* × *C. prunifera* (75; Fig. 6b). The species that shared the lowest number of conversions belonged to *Acrocomia* and *Copernicia*: *C. prunifera* × *A. intumesces* (54), *C. prunifera* × *A. totai* (54), *C. alba* × *A. intumescens* (54), and *C. alba* × *A. totai* (54). The *ndh* genes were the ones with the highest rate of changes, 38 in total (see Supplementary Table S3). However, except for the *ndhD* and *ndhF* genes, the modifications were the same for all six palms. The *matK* gene showed 11 conversions in RNA editing sites, considering the six palms. Even without considering the changes caused by alignments of the chloroplast genomes, it was observed that one conversion was exclusive to *Acrocomia* species and another to *Copernicia* palms. Similarly, two conversions were not exclusively detected in these species.

### Phylogenomic studies

**Figure 7:**
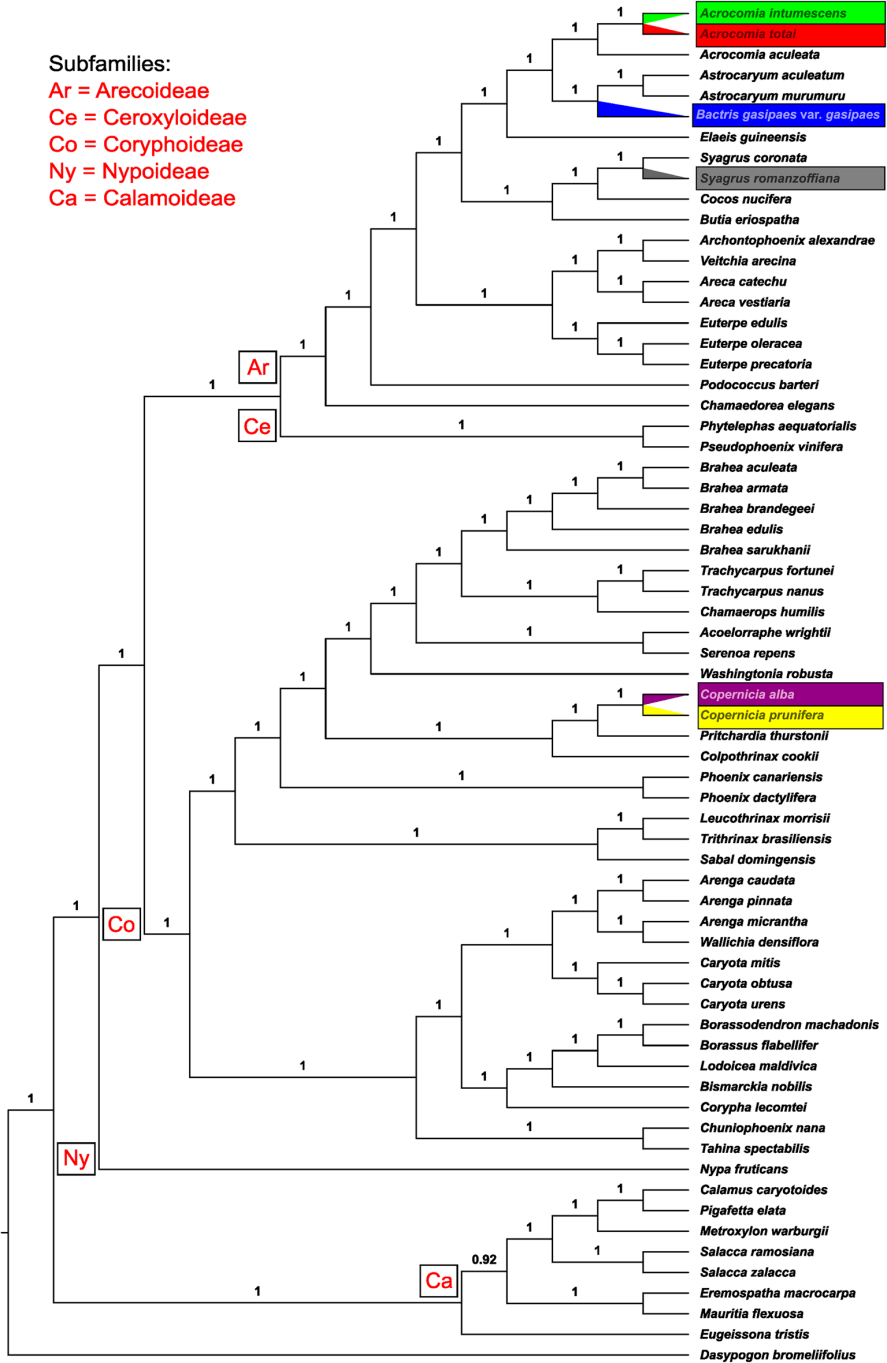
Majority-rule consensus tree of 30,000 trees obtained from a Bayesian inference analysis of chloroplast protein-coding genes of 66 taxa. Posterior probabilities (PP) for each are indicated above the branches.

The full phylogenetic analysis with the selected partition scheme produced a tree (Fig. 7) in which all nodes have a posterior probability of 1.0 (PP = 1.0), except the node that represents the sister relationship between the clade with *Mauritia flexuosa* + *Eremospatha macrocarpa* Schaedtler and the clade containing *Salacca* Reinw.*, Metroxylon* Rottb.*, Pigafetta* (Blume) Becc.*,* and *Calamus* L. (PP = 0.92)*.* Regarding the species with new chloroplast genome sequences generated in the current study,* A. intumescens* and *A. totai* were sister to each other, and then sister to *A. aculeata*.* B. gasipaes* var. *gasipaes* was positioned as sister to two species of *Astrocaryum*, and* S. romanzoffiana* was sister to the previously sequenced *S. coronata* (Mart.) Becc. The species of *Copernicia* were sister to each other and positioned in a small clade, with *Pritchardia* Seem. & H. Wendl. and *Colpothrinax* Griseb. & H. Wendl. being more closely related to the former.

## Discussion

Considering the subfamily level, significant conservation in the genetic content and genomic structures of the chloroplast genome in the analyzed species was observed. The chloroplast genome of *B. gasipaes* showed a small variation in total size from the chloroplast genome already published^35^, from 156,646 to 155,078 bp. This result can probably be caused by differences in the origin and domestication status of the sampled individuals of *B. gasipaes*. The chloroplast genome assembled by da Silva et al.^35^ was from a wild variety, *Bactris gasipaes* var. *chichagui*. In contrast, the individual of *B. gasipaes* var. *gasipaes* used in our research was domesticated and of Peruvian origin. It is also important to highlight that the sequencing method and assembly program applied to each of the varieties were different, which may also result in variations in the assembly size, as already observed for species from the genus *Euterpe*^36^. Thus, more extensive sampling, together with the information provided by the assembly of chloroplast genomes, may provide support for distinguishing individuals of different origins and domestication.

Multiple alignments with 24 different palms (Supplementary Fig. S1) from all five subfamilies did not demonstrate any major rearrangements in the chloroplast structure. The only rearrangement observed was a 4.6 kb inversion in the *Astrocaryum* chloroplast genome (Fig. 2), which is probably lineage-specific^32^. Regarding the patterns of IR structure, most of the expansion/contraction-related variations in the six new chloroplast genomes were identified. It has been previously suggested that chloroplast DNA comprises four equimolar isomers, with the LSC potentially exhibiting either the same or opposite direction. Additionally, variations at the edges of each chloroplast genome region may arise from chloroplastidial DNA replication^37,38^. The most notable difference was with the species *S. romanzoffiana*. This species had a reduced copy of the *rps19* gene compared to the other species of the Arecoideae subfamily (Table 1, Fig. 3), now classified as a fragment. Two hypotheses can be proposed concerning this change: the first hypothesis suggests that this fragmentation event was a consequence of changes induced by the replication mechanism of the chloroplast genome^37^, but without affecting the functional role of the *rps19* gene copy. However, an alternative hypothesis has been proposed regarding the assembly of the complete chloroplast genome of *Cocos nucifera* L*.*^35^. In this species, the presence of a *rps19* fragment was considered a putative pseudogene. Recently, in the chloroplast genome of *Butia eriospatha* (Mart. ex Drude) Becc., the absence of the duplicated *rps19* gene was also observed^2^. Although not discussed, this same event was likewise found in the complete chloroplast genome of *Syagrus coronata*^30^, which, like *S. romanzoffiana*, showed a copy of *rps19* with the same size, 193 bp. The four species are closely related, as a phylogeny based on chloroplast genome sequences identified that *C. nucifera*, *S. coronata*, and *B. eriospatha* shared the same branch and can be classified as sisters in the subtribe Attaleinae of the tribe Cocoseae^35^. Considering the close phylogenetic relationship between the species, such a process should be further investigated.

Although they are considered highly conserved structures for stabilizing chloroplast genome structure^39^, fluctuations caused by contractions/expansions of the IRs have already been reported in palms^31,36^. The most surprising case was the total loss of one IR in the species *Tahina spectabilis* J.Dransf. & Rakotoarin., instead of rearrangement, as this structure was considered canonical in monocots^29^. Since it was a minor fluctuation in IR regions, the reduction of *rps19* in *C. nucifera, B. eriospatha*, and *Syagrus* species can be considered moderate in evolutionary terms. However, given that this was a deviation from a highly conserved structure, this information helps to reinforce the perspective raised by Barret et al.^29^, that these changes may be more prevalent than was previously hypothesized, given that the same change was detected in different species of the same subtribe.

As for the number of SSRs, they were found to be quite conserved among species of the same genus. Besides the number, the distribution of SSR types (mono-, di-, tri-, tetra, penta-, and hexanucleotides; Fig. 4) was highly similar, especially the mono- and dinucleotides. In all species, the mononucleotide A/T was the most frequent motif. This characteristic was also reported in other palm complete chloroplast genomes^31,32,36^. Plastid SSR markers, especially when combined with nuclear markers, can improve the efficiency of studies of structure, diversity, and gene flow in natural populations^40^. Furthermore, plastid SSRs are efficient markers for differentiate highly related species. Variations in the numbers of SSRs based on the chloroplast genomes of *Ceriops* species (Rhizophoraceae) reinforced the differences between *C. tagal*, *C. decandra,* and *C. zippeliana*^25^. Thus, the sequences identified are a valuable resource for knowledge leveling and sustainable planning for species management.

Similarities in the number and distribution of dispersed repeats were also observed for *A. intumescens, A. totai*, and *B. gasipaes* var. *gasipaes*. These parallels could be detected in both the number and distribution of repetition types (Fig. 5). *S. romanzoffiana* showed a higher number of reverse than forward repeats, unlike the first three species. In contrast, the species that differed the most were those from the genus *Copernicia*. The two species differed in both the distribution and size of the dispersed repeats compared to the other species belonging to the subfamily Arecoideae. In particular, the two species of *Copernicia* also differed from each other.

Although at a lower frequency, different numbers of dispersed repeats have already been reported in other land plants. In trees of the genus *Morus* L. (Family: Moraceae), only 14 dispersed repeats were detected, and of these, the species *M. multicaulis* Perr. and *M. cathayana* Hemsl. shared only four^41^. Among seven species of the genus *Polystachya* Hook., there were a variation of two (*P. dendrolliflora* Rchb.f.) and eight (*P. modesta* Rchb.f.) dispersed repeats. Variations in these repetitions may indicate flexibility in the evolutionary process^42^, and this may be considered an indicator of diversity among species, which is important for the phylogeny of the genus^41^.

The post-transcriptional process originated from RNA editing may induce the occurrence of substitutions or indels, which can result in transcript alterations. Editing events usually increase the hydrophobicity of encoded amino acids. In general, the conversions are from serine to leucine/phenylalanine (hydrophilic to hydrophobic) and frequently appear in interfaces to benefit interactions^39,43^. As identified in the six palms, an example of a hydrophobic benefit was observed in the *ndh* complex genes, which encode membrane-binding polypeptides responsible for the transfer of NADH to plastoquinone. The resulting level of hydrophobicity of the new RNA editing sites increases the stability of these trans-membrane proteins^43^. The *matK* gene is known for its rapid evolution and has been a favorite for determining phylogenetic relationships in angiosperms^44^. With the chloroplast genome of the six palms, it was possible to observe the dynamics of the RNA editing sites in this gene and identify the appearance of unique conversions in certain species. Some authors have already reported that the rapid evolution of *matK* creates a selective pressure favoring C-to-T mutations, which reflects in the loss of RNA editing sites^44^. The addition of new chloroplast genomes might clarify what is leading to the loss or appearance of these sites.

Despite a few exceptions, RNA editing is evolutionary conserved, and it is expected that more related taxa will have more editing sites in common^43^. Of the 102 RNA editing sites, 54 were shared among the six palm species (Fig. 6a). Also, species from the same subfamily have more RNA editing sites in common (Coryphoideae: 20 and Arecoideae: 19; Fig. 6b). In addition to the subfamily hierarchy, the species *A. intumescens, A. totai, B. gasipaes*, and *S. romanzoffiana* are from the same tribe, Cocoseae, and the species from the genera *Acrocomia* and *Bactris* also participate in the same subtribe, Bactridinae^41^. This was reflected in the fact that they shared more than 70 RNA editing sites. Similarly, the species of the genus *Copernicia*, being phylogenetically close, shared 75 RNA editing sites.

In the phylogeny by Meerow et al.^46^, based on WRKY nuclear genes, *Acrocomia* was sister to *Astrocaryum* and then both to *Bactris* + *Desmoncus*, however with low support, and both clades in a polytomy with *Aiphanes* Willd. There is no chloroplast genome available for either *Desmoncus* Mart. or *Aiphanes*, but our phylogeny indicates with PP = 1.0 that *Bactris* is sister to *Astrocaryum* and both to *Acrocomia* (Fig. 7). This relationship is also in agreement with the whole chloroplast genome phylogeny of Silva et al.^35^, which included their sequence of the *Bactris* chloroplast genome, and the phylogenomic supertree-based study of Barret et al.^29^. The relationship among the *Acrocomia* species indicated *A. totai* as sister to *A. intumescens* and then both sisters to *A. aculeata.* This is also supported by the phylogenetic and biogeographic studies with different evolutionary models showing that *Acrocomia* and *Aiphanes* were the first Bactridinae genera to irradiate in the late Eocene before the final uplift of the Andes (late Miocene and Pliocene)^47^. In the population genomics study based on Genotyping by Sequencing data from Díaz et al.^10^, *A. totai* was closer to *A. aculeata* (in a genetic distance dendrogram), whereas *A. intumescens* was more divergent in relation to this pair. On the other hand, our results reaffirm the close relationship between *A. aculeata* and *A. intumescens*, also found by Meerow et al.^46^, and with leaf anatomy by Vianna et al.^48^, although some authors still considered the species synonyms^5,49^. It is important to point out that *A. aculeata* has the widest distribution among the Neotropical palms, from central Mexico to northern Argentina^10^. *Acrocomia* still has unshed phylogenetic relationships due to its hybridization process, domestication, and possible ecotypes because a complete genus phylogeny is not yet available, including a significant number of botanical holotypes and natural samples comprising all its distribution.

Finally, the position of *Syagrus romanzoffiana* with *S. coronata* is expected, and its sister relationship to *Cocos* and then both to *Butia* agrees with the Cocosoid Palms by Meerow et al.^46^, and the phylogenomic study of Barret et al.^29^. The position of *Copernicia* is quite distant from the previous terminals since they belong to the subfamily Coryphoideae. Our phylogeny indicated a sister relationship between *Copernicia* spp. and *Pritchardia*, and between both and *Colpothrinax*, similar to Barret et al.^29^.

## Conclusion

Among the six Neotropical palms studied, gene content and chloroplast genome structure were highly conserved. Some punctual changes were observed, such as different tRNAs in *Copernicia* palm species in relation to the other four palms from the subfamily Arecoideae. Multiple alignments with palms from all Arecaceae subfamilies revealed no major rearrangements in chloroplast structure. Regarding the IRs, most of the variations were expansion/contraction. A reduction of the *rps19* gene copy was observed in *S. romanzoffiana*, a moderate but prevalent change in evolutionary terms, as it was identified in other species of the tribe Cocoseae. Conservation in the number of SSRs was also detected in palms of the same genus. However, some motifs occurred only in *B. gasipaes* var. *gasispaes* and *S. romanzoffiana*. The sequences identified are valuable for obtaining genetic markers. Similarities in the number and distribution of dispersed repeats were observed in the subfamily Arecoideae. Nevertheless, these repeats presented a distinct pattern in *Copernicia* palms, considered an indicator of diversity. For RNA editing, it was observed that related taxa had more sites in common, as expected. Finally, our phylogeny presented high support, and the positioning of the six palms was like those found in previous studies. Especially in the case of the genus *Acrocomia*, our results restated the close relationship between *A. aculeata* and *A. intumescens*. Overall, a highly consolidated pattern was identified among palm chloroplast genomes, with subtle structural changes. Resources were provided for evolutionary analyses of the Arecaceae family as well as for species conservation studies.

## Methods

### Species, sampling, and DNA extraction

To characterize the geographical occurrence of the six palm species, we surveyed the databases using the BIEN^51^ R package^52^. *Bactris gasipaes* is dispersed in Central and South America, while *Acrocomia totai*, *Copernicia alba*, and *Syagrus romanzoffiana* occur in South America. *Acrocomia intumescens* and *C. prunifera* have records only in the Brazilian Northeast Region (see Supplementary Fig. S3). All palms in this study were wild plants, except for *B. gasipaes*. This palm was an introduction of domesticated seed from Yurimáguas, Peru, in the 1970s, also known as *Bactris gasipaes* var. *gasipaes*.

The leaves were dried with silica gel and stored in a freezer at − 20 °C. The leaf material from *A. intumescens, A. totai, B. gasipaes* var. *gasipaes*, *C. alba*, and *C. prunifera* was obtained in the active germplasm bank of the Plant Genetic Resources Center of the Agronomic Institute of Campinas (IAC), Campinas, SP, Brazil (geographical coordinates: − 22.8717, − 47.0776). *S. romanzoffiana* was sampled from the ex situ collection at the IAC in Piracicaba, Piracicaba-SP, Brazil (geographical coordinates: − 22.6836, − 47.6458). This study complies with relevant institutional, national, and international guidelines and legislation. The appropriate permissions for the collection of plant material were taken, and the collections were registered according to the National System for the Management of Genetic Heritage and Associated Traditional Knowledge (SISGEN), as stated by Brazilian Decree No. 8,772 (May 11, 2016) and regulated by Brazilian Law No. 13,123 (May 20, 2015; SISGEN numbers: A411583 and A9BEE40, Brazil). The voucher numbers are shown in Supplementary Table S4 and are available at the IAC Herbarium.

To extract the chloroplast organelles, a sucrose gradient method was used to isolate them^53^. For this, 20 g of fresh leaves from each species were frozen with liquid nitrogen and macerated. The material was resuspended in 200 mL of isolation buffer (50 mM Tris–HCl pH 8.0, 0.35 M sucrose, 7 mM EDTA, 5 mM 2-mercaptoethanol, and 0.1% BSA) and incubated for 10 min in the dark. The suspension was filtered using two layers of Miracloth (Merck), and then the filtrate was centrifuged at 1000×*g* for 10 min.

The pellet was resuspended in 5 mL of isolation buffer. The suspension was placed in the density gradient column of 20/45% sucrose in 50 mM Tris–HCl (pH 8.0), 0.3 M sorbitol, and 7 mM EDTA. After the centrifugation at 2000×*g* (30 min), the green band formed at the interface containing intact chloroplasts was collected. The solution with the chloroplasts was then diluted in three volumes of buffer and centrifuged at 3000×*g* (10 min) to obtain a pellet with purified chloroplasts.

The pellet was then resuspended in 2% CTAB buffer to initiate lysis. The suspension was incubated and stirred at 65 °C for 1 h. The supernatant was extracted twice with an equal volume of chloroform: isoamyl alcohol (24:1) and centrifuged at 10,000×*g* (20 min). The same volume of isopropanol was added and incubated at 20 °C for 1 h. Lastly, the aqueous phase was centrifuged at 10,000×*g* (20 min). The chloroplast DNA (cpDNA) pellet was washed with ethanol (70%), dried, and resuspended with 40 µL TE (1 M Tris–HCl, 0.5 M EDTA, pH 8).

### Chloroplast genome sequencing, assembly, and annotation

The genomic libraries were constructed using 100 ng of cpDNA and the Nextera DNA Flex kit (Illumina), following the manufacturer's instructions. Paired-end sequencing (2 × 150 bp) was performed on the Illumina NextSeq550 platform (Fundação Hemocentro de Ribeirão Preto, Brazil).

Two programs were used to assemble the complete chloroplast genomes of the six Neotropical palms, as they presented different benefits. First, NOVOPlasty was applied to all palms, as its main advantage was the fast de novo assembly of organelle genomes (chloroplasts and mitochondria) from unfiltered whole genome sequence (WGS)^54^. This resulted in high-quality genomes in terms of coverage and accuracy^54^. However, raw WGS-based assembly is not always successful. Many sequencing technologies can result in error-prone, such as highly repetitive regions. With this, NOVOPlasty can generate multiple contigs^54^, making it difficult to circularize the chloroplast genome, this was the case of *B. gasipaes*. Therefore, we adopted another strategy as a way to reduce the ratio of nuclear reads in relation to organelle reads. Before assembly, we mapped them onto a reference genome and performed their filtering^54^. This process required more pre-assemble steps, however, with the partial extraction of data from the original files, it was possible to use NOVOWrap^55^. The program is partly based on NOVOPlasty, but its main advantage is a more automated process with the identification and testing of different seeds according to related organisms present in the literature. Testing different seeds and reference genomes provides additional opportunities for successful assembly^55^. However, this kind of assembly requires high computational memory, which can be a limiting factor.

In this way, the chloroplast genome assembly of *Acrocomia intumescens*, *A. totai, Copernicia alba, C. prunifera*, and *Syagrus romanzoffiana* was performed in two steps: Firstly, the paired-end reads from these five palms were assembled in NOVOPlasty v4.2^54^ (https://github.com/ndierckx/NOVOPlasty) using the *rbcL* gene sequence as a seed (NCBI accession numbers: for *A. intumescens* and *A. totai*, *rbcl* from *A. aculeata*: AY044625.1; *rcbl* of *C. prunifera*: AM110199.1; *rbcl* of *C. alba*: MK753471.1; and *rbcl* of *S. romanzoffiana*: GU135249.1), and the chloroplast genome of *Acrocomia aculeata* (NCBI accession number: NC_037084.1) as a reference to order the contigs^56^. Secondly, the confirmation of quality, correctness, and coverage of the assembly was carried out using Geneious v2020 2.4 (https://www.geneious.com/, last assessed January 2022). We applied the “Map to reference” function to map the paired-end raw data onto the final assembled chloroplast genomes.

Using BWA and SAMTools^57,58^, the sequences from the chloroplast genomes of other palms were indexed (Supplementary Table S4) and mapped with the raw reads of *B. gasipaes* var. *gasiapes* to filter out the ones present only in the organelle genome. After that, the output files .bam were converted to .fastq with BEDTools^59^. With the filtered.fastq files, the chloroplast genome assembly was performed with NOVOWrap v1.20^55^, using the *psaC* gene as seed (NCBI accession number: MH537788) and *Astrocaryum aculeatum* (NCBI accession number: MH537788) as reference genome to order the contigs. Finally, Geneious v2020 2.4 (https://www.geneious.com/, last assessed January 2022) was used, as previously described. The raw reads for each species, as well as the coverage resulting from the assembly of each chloroplast genome, are available in Supplementary Table S4.

The annotation of the chloroplast genomes was performed in GeSeq (Organellar Genome Annotation)^60^ from the Chlorobox platform, with settings for the identification of protein coding sequences (CDS), rRNAs, and tRNAs based on reference chloroplast sequences and homologies through BLAST search. Following the GeSeq annotation, GenomeView^61^ was used to conduct a manual correction of start and stop codons and verify pseudogene and intron positions. We then obtained the chloroplast circular genome maps using OGDRAW^62^.

### Chloroplast genome structure comparison

We conducted two multiple progressive sequence alignments in Mauve v2.4.0^63^. The first one included the six new chloroplast genomes and those that were available in GenBank from species that occur in Brazil: *Acrocomia aculeata*, *Astrocaryum aculeatum*, *A. murumuru, Butia eriospatha, Euterpe edulis* Mart., *E. oleracea* Mart., *E. precatoria* Mart.*, Mauritia flexuosa, Syagrus coronata*, and *Trithrinax brasiliensis.* The second analysis was carried out using 24 chloroplast genomes from different palm species (Supplementary Table S5). We selected species that represented the five palm subfamilies, considering the evolution of the group: *Phytelephas aequatorialis* Spruce and *Pseudophoenix vinifera* (Mart.) Becc. (Subfamily: Ceroxyloideae); *Copernicia alba, C. prunifera, Caryota mitis* Lour., *Trachycarpus fortune* (Hook.) H. Wendl., and *Trithrinax brasiliensis* (Subfamily: Coryphoideae); *Nypa fruticans* (Subfamily: Nypoideae); *Calamus caryotoides* A. Cunn. ex Mart., *Eremospatha macrocarpa* Schaedtler, and *Mauritia flexuosa* (Subfamily: Calamoideae); *Veitchia arecina* Becc. (Subfamily: Arecoideae); and the Brazilian native species also from the Arecoideae subfamily (Supplementary Table S5).

The six chloroplast genomes also presented contractions and expansions in the inverted repeat (IR) regions. Since these regions may show structural differences, it is expected to identify variability among species and within palm subfamilies (Arecoideae: *Acrocomia intumescens, A. totai, Bactris gasipaes*, and *Syagrus romanzoffiana;* Coryphoideae: *Copernicia alba* and *C. prunifera*)*.*

### Identification of SSRs and dispersed repeats

Single sequence repeats (SSR) containing 1–6 nucleotides were identified using the MISA web package (available at https://webblast.ipk-gatersleben.de/misa/)^64^. To search for SSR motifs, the following configuration was considered: SSR of one to six nucleotides long, with a minimum repeat number of 10, 5, and 4 units for mono-, di-, and trinucleotide SSRs, respectively, and three units for tetra-, penta-, and hexanucleotide SSRs. The determination of dispersed repeats (forward, reverse, palindrome, and complement sequences) was performed in REPuter (available at: https://bibiserv.cebitec.uni-bielefeld.de/reputer)^65^ based on the following criteria: minimum repetition size ≥ 30 bp and sequence identity ≥ 90% (Hamming distance = 3). The composition and position of the SSRs and dispersed repeats were manually compared among each chloroplast genome.

### RNA editing sites

The RNA editing sites of the chloroplast genomes of *A. intumescens, A. totai, B. gasipaes* var. *gasipaes, C. alba, C. prunifera*, and *S. romanzoffiana* were predicted using predictive RNA editor for plants (PREP)^66^. For this, 35 coding sequences from each chloroplast genome and a cutoff value of 0.8 were used. RNA editing sites were compared between species, considering their positions as well as their amino acid substitutions.

### Phylogenomic studies

The annotated GenBank files of each species were imported using a set of Python scripts developed in our group (available under request from CvdB) into a SQLite database. All the putative coding regions were extracted, and each region was individually aligned using MUSCLE v5.1^67^. Then, all the aligned regions were concatenated into a Nexus file, including ‘charsets’ for each individual region and for each codon position in each region. The regions *cemA*, *ndhD*, *petD*, and *rps12* did not produce alignments compatible with codon assignment (missing start codons, non-ternary indels, etc.) and were therefore separated from the canonical coding regions for further testing. Evolutionary models were assessed using four different partition schemes: (i) two partitions, with one model for all coding regions versus a different model for all non-canonical regions (*cemA*, *ndhD*, *petD*, and *rps12*); (ii) five partitions, with one model for all coding regions and four different models for *cemA*, *ndhD*, *petD*, and *rps12*; (iii) four partitions, with three different models for each codon position (1st, 2nd, and 3rd) versus one model for all non-canonical regions; and iv) seven partitions, with three different models for each codon position (1st, 2nd, and 3rd) and four different models for *cemA*, *ndhD*, *petD*, and *rps12*. The evolutionary models for each partition in the partition schemes were estimated with MrModeltest v2.4^68^, and then the different partition schemes were assessed using Bayes Factors^69^ after four Stepping-Stone (SS) analyses of each scheme using MrBayes v3.2.7^70^. Each analysis consisted of two runs with four chains each (one cold and three hot chains) for 20 million generations and a burn-in of 25%. The marginal likelihood of each analysis was estimated using a stepping-stone sampling of 50 steps with 196,000 generations. For phylogeny estimation of the best-selected partition scheme, MrBayes was then run with the same number of runs and chains, 20 million generations, one tree sampled every 1000 steps, and 25% burn-in. After checking convergence and ESS > 200 (in fact, no parameter was under 8000), the majority-rule consensus was used as an estimation of the phylogeny and posterior probabilities.

The assessment of the different partition schemes indicated a large difference in scheme (iii), with an average marginal log-likelihood (AML) of − 189,272.26, against model (i) =  − 191,019.93, model (ii) =  − 191,092.66, and model (iv) =  − 189,485.41. The difference between the two best models (iii–iv) was 213.15, which indicated very strong evidence under the criterion of Bayes Factors^69^, for the scheme with four partitions (a model for each codon position and a single model for the regions that could not have codons properly assigned, putatively non-coding).

### Supplementary Information

Supplementary Figures.

Supplementary Tables.

## Data Availability

The datasets generated and analysed during the current study are available in the NCBI's GenBank repository (https://www.ncbi.nlm.nih.gov/), with the accession numbers and direct links: *Acrocomia intumescens* (OQ129926; https://www.ncbi.nlm.nih.gov/nuccore/OQ129926), *Acrocomia totai* (OQ129927; https://www.ncbi.nlm.nih.gov/nuccore/OQ129927), *Bactris gasipaes* (OQ129928; https://www.ncbi.nlm.nih.gov/nuccore/OQ129928), *Copernicia alba* (OQ129929; https://www.ncbi.nlm.nih.gov/nuccore/OQ129929), *Copernicia prunifera* (OQ129930; https://www.ncbi.nlm.nih.gov/nuccore/OQ129930) and *Syagrus romanzoffiana* (OQ129931; https://www.ncbi.nlm.nih.gov/nuccore/OQ129931).
