# Supplementary material for: Complete chloroplast genomes of six neotropical palm species, structural comparison, and evolutionary dynamic patterns
Source: Sci Rep. 2023 Nov 23;13:20635. doi: 10.1038/s41598-023-44631-4 (PMC10667357; doi:10.1038/s41598-023-44631-4)
Supplement: Supplementary file 1 — Supplementary Figures. [file 41598_2023_44631_MOESM1_ESM.pdf]

Supplementary Material - Figures

Complete chloroplast genomes of six Neotropical palm species, structural comparison, and evolutionary dynamic patterns

Ana Flávia Francisconi, Jonathan Andre Morales Marroquín, Luiz Augusto Cauz dos Santos, Cássio van den Berg, Kauanne Karolline Moreno Martins, Marcones Ferreira Costa, Doriane Picanço-Rodrigues, Luciano Delmodes de Alencar, Cesar Augsuto Zanello, Carlos Augusto Colombo, Brenda Gabriela Díaz, Danilo Trabuco Amaral, Maria Teresa Gomes Lopes, Elizabeth Ann Veasey, Maria Imaculada Zucchi

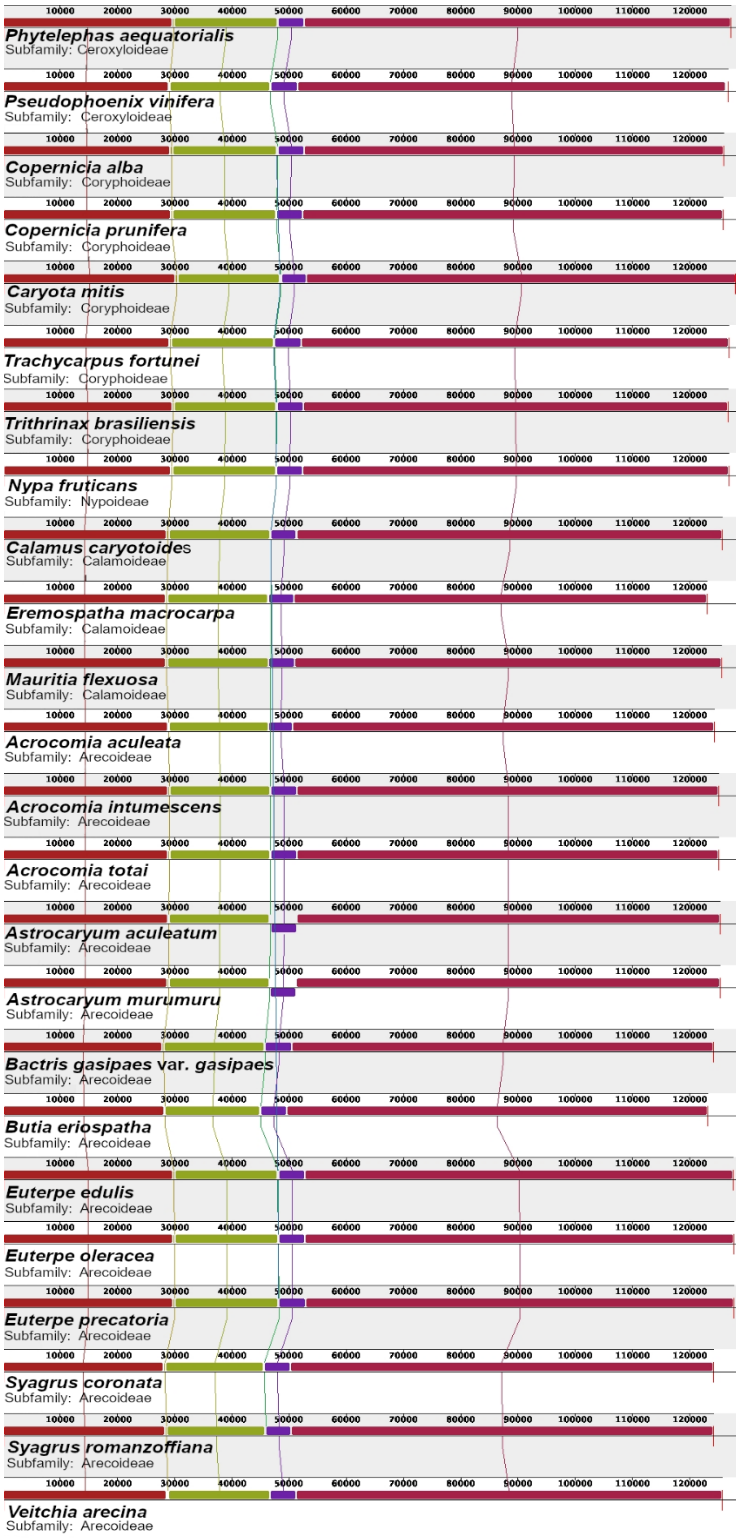

Figure S1. Synteny detected in 24 Arecaceae chloroplast genomes using the Mauve multiple-genome alignment program. Color bars indicate syntenic blocks, and the lines indicate the correspondence between them. Blocks on the top row are in the same orientation, while blocks on the bottom row are in inverse orientation.

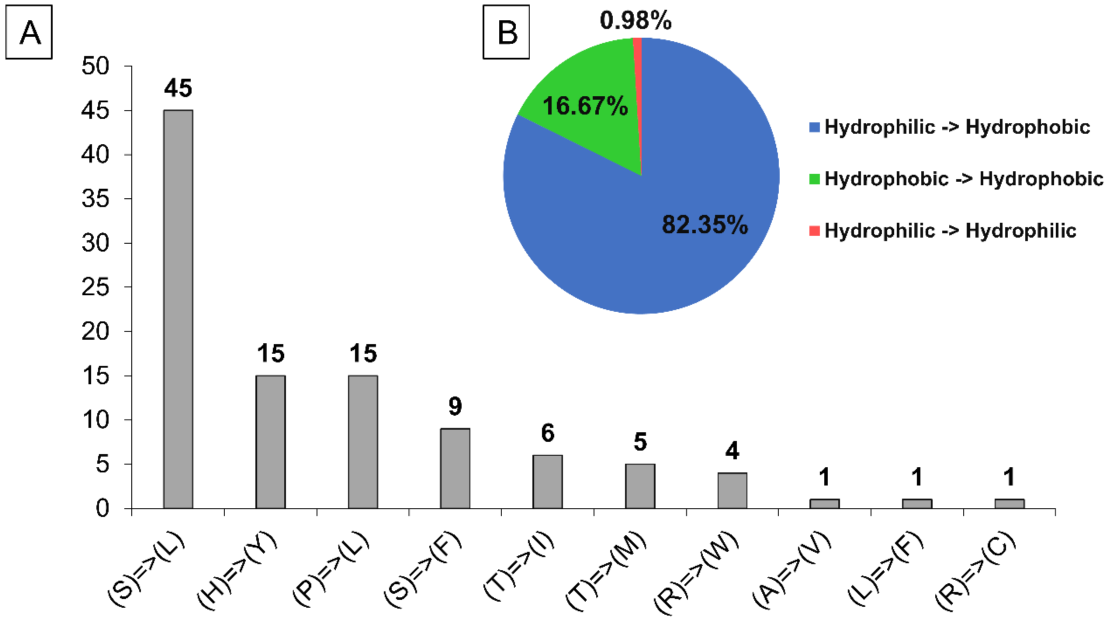

Figure S2. Conversions presented in the RNA editing sites of the species *Acrocomia intumescens, A. totai, Bactris gasipaes, Copernicia alba, C. prunifera*, and *Syagrus romanzoffiana*. (A) Number of conversions according to amino acid changes in RNA editing sites shared among the six species; (B) Percentage of polarity change caused by amino acid conversions at RNA editing sites found between them.

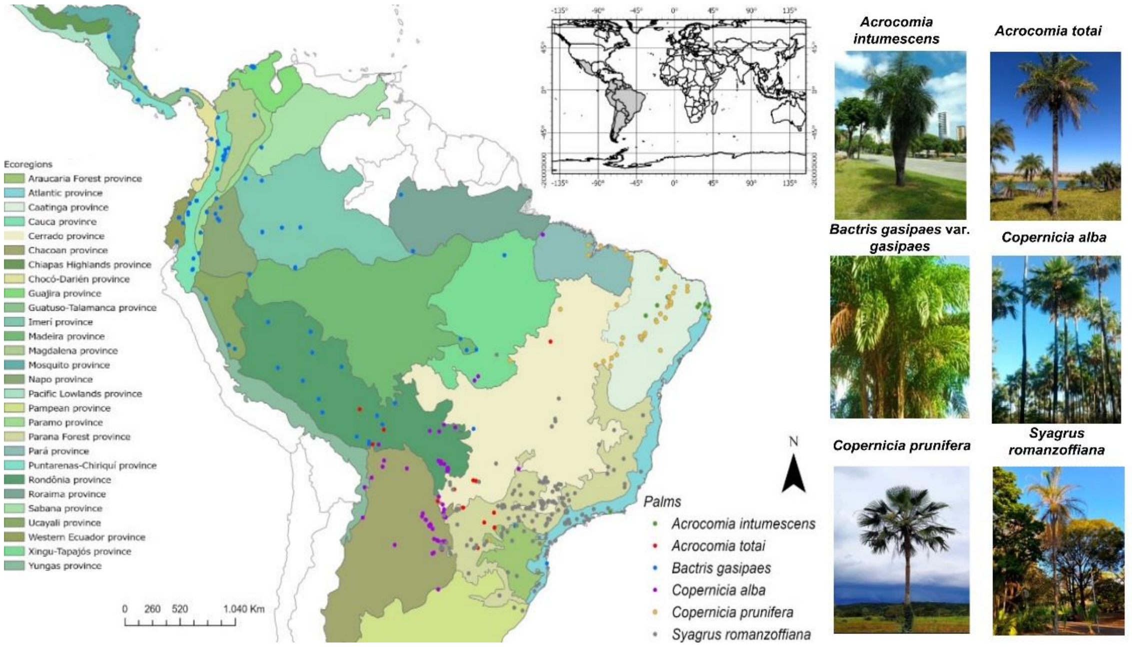

Figure S3. Distribution of the species *Acrocomia intumescens*, *A. totai*, *Bactris gasipaes*, *Copernicia alba*, *C. prunifera*, and *Syagrus romanzoffiana* according to online herbarium databases. Photos taken by the authors: *A. intumescens* and *A. totai* (BGD); *B. gasipaes* var. *gasipaes* (DPR); *C. alba* and *C. prunifera* (MFC); and *S. romanzoffiana* (AFF). Shapefile available at https://neotropicalmap.atlasbiogeografico.com/ [1].

References

1. Morrone, J. J., T. Escalante, G. Rodríguez-Tapia, A. Carmona, M. Arana & J. D. Mercado-Gómez. 2022. Biogeographic regionalization of the Neotropical region: New map and shapefile. *An. Acad. Bras. Cienc.* **94(1),** e20211167 (2022). https://10.1590/0001-3752022220211167
